# A Portable, Inexpensive, Nonmydriatic Fundus Camera Based on the Raspberry Pi® Computer

**DOI:** 10.1155/2017/4526243

**Published:** 2017-03-15

**Authors:** Bailey Y. Shen, Shizuo Mukai

**Affiliations:** ^1^Department of Ophthalmology, Illinois Eye and Ear Infirmary, University of Illinois at Chicago, 1855 West Taylor Street, Chicago, IL 60612, USA; ^2^Retina Service, Massachusetts Eye and Ear Infirmary, Harvard Medical School, 243 Charles Street, Boston, MA 02114-3096, USA

## Abstract

*Purpose.* Nonmydriatic fundus cameras allow retinal photography without pharmacologic dilation of the pupil. However, currently available nonmydriatic fundus cameras are bulky, not portable, and expensive. Taking advantage of recent advances in mobile technology, we sought to create a nonmydriatic fundus camera that was affordable and could be carried in a white coat pocket. *Methods.* We built a point-and-shoot prototype camera using a Raspberry Pi computer, an infrared-sensitive camera board, a dual infrared and white light light-emitting diode, a battery, a 5-inch touchscreen liquid crystal display, and a disposable 20-diopter condensing lens. Our prototype camera was based on indirect ophthalmoscopy with both infrared and white lights. *Results.* The prototype camera measured 133mm × 91mm × 45mm and weighed 386 grams. The total cost of the components, including the disposable lens, was $185.20. The camera was able to obtain good-quality fundus images without pharmacologic dilation of the pupils. *Conclusion.* A fully functional, inexpensive, handheld, nonmydriatic fundus camera can be easily assembled from a relatively small number of components. With modest improvements, such a camera could be useful for a variety of healthcare professionals, particularly those who work in settings where a traditional table-mounted nonmydriatic fundus camera would be inconvenient.

## 1. Introduction

Evaluation of the fundus is an essential component of an eye examination, providing valuable diagnostic information to both ophthalmologists and nonophthalmologists. In addition to physical examination, the fundus can also be photographed, which allows for documentation and sharing of the images for telemedicine. Our group has recently published on using smartphones combined with handheld indirect lenses for mydriatic fundus photography [1]. This technique offers the advantages of being both inexpensive and portable compared to most current methods of fundus photography.

Most fundus photography techniques, including our smartphone system described above, require pharmacologic dilation of the pupil. Pharmacologic dilation, while a routine and critical part of standard ophthalmic practice, has some significant disadvantages. First of all, nonophthalmologists tend to be unfamiliar with using dilating drops. In addition, regardless of specialty, pharmacologic dilation tends to be inconvenient for both the medical practitioner and the patient, with the dilating drops taking about twenty minutes to take effect and the patient experiencing blurred vision and light sensitivity for up to several hours after dilation. Furthermore, pharmacologic dilation prevents subsequent physical examination of the pupils for several hours, an effect that can be undesirable when monitoring inpatients with critical neurologic disease. Finally, there is a small but real risk of inciting acute angle-closure glaucoma in susceptible eyes with the use of dilating eye drops [2].

Nonmydriatic fundus photography allows for imaging of the retina and optic nerve without pharmacologic dilation [3]. There are different ways in which this can be done, but one common method is to use infrared light, to which the pupil does not constrict, to focus on the fundus, and then to quickly flash white light to capture a color fundus photograph before the pupil can constrict [3]. Since the eye does not perceive the infrared light used to focus the camera on the fundus, nonmydriatic photography, even with the white flash, is often more comfortable for the patient compared to physical examination of the fundus with white light in indirect ophthalmoscopy. Unfortunately, currently available nonmydriatic cameras tend to cost thousands of dollars, and most are either table-mounted or too bulky to carry around conveniently [3, 4].

For this project, we sought to take advantage of current camera and light-emitting diode (LED) technology to create a prototype nonmydriatic fundus camera that was affordable and small enough to carry in a white coat pocket. Such a camera could be used in a variety of settings, including outpatient and inpatient areas, emergency departments, and screening clinics, and could potentially be useful for telemedicine.

## 2. Methods

### 2.1. Hardware

Our nonmydriatic fundus camera is based on the Raspberry Pi 2 Model B and the NoIR camera board (Raspberry Pi Foundation, Caldecote, Cambridgeshire, UK). The Raspberry Pi is a credit card-sized computer board designed to easily interact with its environment. The NoIR camera board is a 5-megapixel camera, similar to consumer smartphone cameras except that it is sensitive to infrared light and is fixed-focus. For our fundus camera, we changed the focus of the stock NoIR camera board from infinity to about 8 cm by unscrewing the lens counterclockwise with two pairs of jewelry pliers [5].

**Figure 1:**
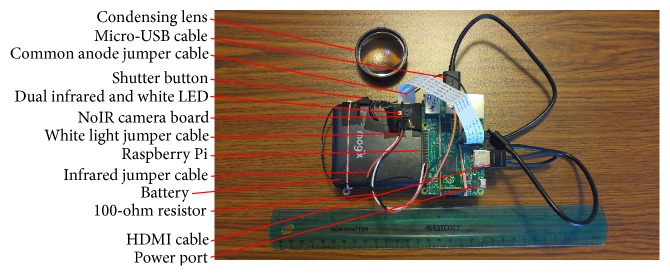
Front of the prototype fundus camera.

**Figure 2:**
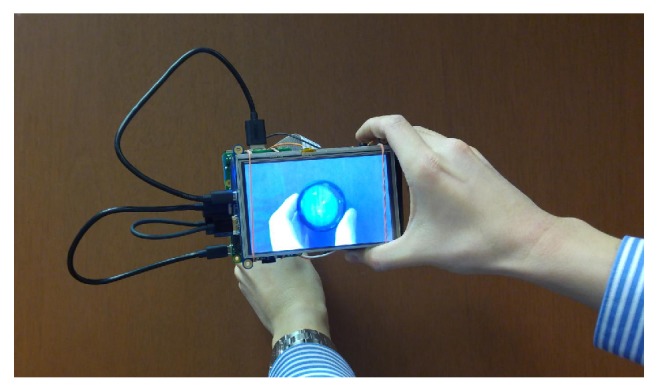
Back of the prototype fundus camera, showing the touchscreen.

The Raspberry Pi is attached to a lightweight 10400 mAh lithium battery and a 5-inch LCD touchscreen by rubber bands (see Figures 1 and 2). The Raspberry Pi is also connected to the battery by a micro-USB cable and to the LCD touchscreen by a micro-USB cable and HDMI cable. The NoIR camera board is taped to the battery.

Illumination of the retina is provided by a prototype dual LED (SMT47W/850D) manufactured by Ushio Epitex Incorporated (Kyoto, Japan) and distributed by Marubeni Corporation. This 3.0 × 3.5mm dual LED can emit either infrared light (850 nm) or white light, with the infrared and white lights being coaxial. The dual LED is taped paraxially to the NoIR camera lens (see Figure 1) and is connected to the Raspberry Pi's GPIO pins via jumper cables soldered to the dual LED. On top of the camera is a physical shutter button, also connected to the Raspberry Pi's GPIO pins with jumper cables.

A disposable 20-diopter condensing lens (Sensor Medical Technologies, Maple Valley, WA, USA), held in the free hand, is used in conjunction with the fundus camera to perform indirect ophthalmoscopy (see Figures 1 and 2). This is similar to the smartphone fundus photography system previously described [1].

The Supplementary Instructions available online at https://doi.org/10.1155/2017/4526243 provide a step-by-step description of how to build our prototype camera. Also available online is a Supplementary Parts List, which provides a cost breakdown.

### 2.2. Software

The operating system used for the Raspberry Pi is Raspbian, which was preloaded on an 8-gigabyte NOOBS micro-SD card (Raspberry Pi Foundation). Commands are typed into the Raspberry Pi through the LCD touchscreen and the open-source Florence virtual keyboard program. A simple Python program, based on the picamera module written by Dave Jones [6], is used for the fundus camera and is provided in the Supplementary Instructions.

## 3. Results

The prototype nonmydriatic fundus camera and condensing lens can be seen in Figures 1 and 2. The camera measures approximately 133mm × 91mm × 45mm (excluding the cables) and weighs 386 grams. The total cost of the fundus camera and condensing lens is approximately $185.20 (see Supplementary Parts List).

### 3.1. Camera Operation

**Figure 3:**
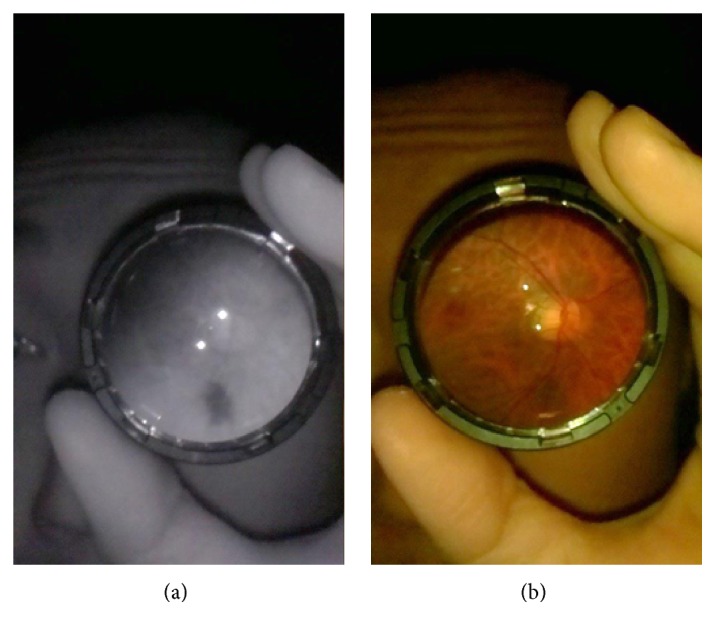
(a) Infrared image of the author's left fundus taken with the prototype fundus camera. (b) Color image of the same fundus.

The fundus camera is turned on by plugging the micro-USB cable into the Raspberry Pi. Once the operating system has loaded, the Python program is started, which turns on the camera's viewfinder as well as the infrared light of the infrared LED. The user of the fundus camera darkens the room, which allows the patient's pupils to naturally dilate. With the fundus camera in one hand, and the condensing lens in the other, the user then performs infrared indirect ophthalmoscopy. This is similar to the mydriatic smartphone fundus photography technique described previously [1]. When the fundus camera, condensing lens, and patient's retina and pupil are properly aligned, the user will see a black-and-white picture of the fundus filling the condensing lens in the viewfinder (see Figure 3(a)). When he or she is satisfied with the image, he or she presses the shutter button, which turns off the infrared LED, flashes the coaxial white LED, and captures a color photograph of the fundus (see Figure 3(b)). The image can then be accessed from the memory card and transmitted to other computers via an Ethernet cable or a wireless dongle connected to the Raspberry Pi.

Figure 3 shows example images taken of one of the author's retinas in low light, with no pharmacologic dilation. The photograph was taken using the “*retina selfie*” technique previously described [7]. The quality of the image seems to be acceptable for making general diagnoses, such as optic disc sharpness. The field of view is about 4 to 5 disc diameters, depending on the distance of the camera to the condensing lens.

### 3.2. Light Intensity

The intensity of the infrared and white light produced by the prototype nonmydriatic fundus camera was measured with a Thorlabs PM100 console and a S120B sensor (Newton, New Jersey, USA). A 20-diopter condensing lens was used to concentrate light on the sensor. When set to infrared, the camera made between 50 and 90 microwatts of light energy at 850 nm. When set to white light, the camera produced between 150 and 170 microwatts of light energy at 570 nm. In comparison, a Keeler Vantage Plus indirect ophthalmoscope (Keeler Ophthalmic Instruments, Broomall, Pennsylvania, USA), when set to half intensity and full intensity, produced around 1500 microwatts and 3000 microwatts of light energy at 570 nm, respectively.

## 4. Discussion

Our prototype camera offers a proof of concept that a portable nonmydriatic fundus camera capable of capturing quality fundus photographs can be produced simply and inexpensively. That such a prototype camera is possible is due in large part to the current smartphone and mobile technology revolution, with improving quality, shrinking sizes, and falling prices of computer chips, camera boards, camera illumination systems, and touchscreens. In fact, as of the creation of our prototype, the Raspberry Pi Foundation has produced an updated version of the Raspberry Pi computer, the Pi 3, with a faster processor and built-in wireless card, and an updated version of the NoIR camera board, the V2, with 8 megapixels of resolution. Both upgraded components are the same price as their previous versions, which were used for our prototype camera.

Our prototype camera also owes its existence to its 3.0 × 3.5mm dual infrared and white light LED, which is capable of producing coaxial infrared and white light. This LED is the only part of our prototype camera that is not available as an off-the-shelf component, but we suspect that this dual infrared and white light LED could be mass-manufactured, and if it were, the price per LED would be around $3 (Marubeni, personal communication). Previously, for illumination of our prototype camera, we had attempted to use a commercially available 5 mm white LED and 5 mm infrared LED taped flush against each other next to the camera lens. Because each 5 mm LED, although small, had a discrete diameter, the infrared and white lights were not sufficiently coaxial for consistent fundus photography. A small dual LED with coaxial infrared and white lights could be an alternative to complex, bulky, and expensive illumination systems found in other nonmydriatic fundus cameras [3, 8].

One limitation of our camera is that since it relies on indirect ophthalmoscopy, there is a learning curve to its use. To operate the camera, users must become facile with maneuvering both the camera and condensing lens so that both are in correct alignment with the patient's pupil. Our camera does not have a stand or patient chin rest as do traditional table-mounted fundus cameras; the lack of a stand or chin rest offers the advantage of portability, but the disadvantage of increased difficulty in maintaining alignment. Nevertheless, the authors feel that ophthalmologists, who frequently use handheld lenses for indirect ophthalmoscopy, could learn how to use our camera adeptly, and this has been our experience with our mydriatic smartphone fundus photography system that uses similar optics and technique [1]. Nonophthalmologists, with practice, may also be able to use our camera. Again, we have been able to teach the mydriatic smartphone system mentioned above to laboratory technicians for fundus photography in rabbits [1]. It is also conceivable that our portable camera could be made modular and “*docked*” to a table-mounted camera stand and chin rest for improved alignment when needed. Additional modifications to the camera, such as weight reduction, autofocus, an automatic shutter, voice command, and a condensing lens holder, may improve its ease of use [9].

Recent work has shown that smartphones can be used for fundus photography [1, 10, 11]. Smartphones have the advantage of being ubiquitous and being easy to connect to mobile or wireless networks. When a smartphone's native camera and flash are used, however, disadvantages of smartphone fundus photography include need for pharmacologic dilation and unknown light safety of the camera flash among different smartphones. In the future, a major possible improvement to our nonmydriatic fundus camera would be to turn the camera into a “*dongle*” for a smartphone, connected to a smartphone via a micro-USB or Lightning® connection. Such a camera dongle would have an infrared-sensitive camera board and dual infrared and white light LED but would rely on the smartphone to provide the battery, touchscreen viewfinder, and internet connection, increasing the portability and decreasing the cost of the camera. An early prototype built in collaboration with colleagues from the Media Lab at the Massachusetts Institute of Technology was presented at ARVO 2016 [12]. This prototype was built at a cost without the phone of approximately $90.

Before use on patients, our prototype camera would require additional light safety testing. Our preliminary results, though, suggest that our prototype camera is likely safe for human eyes. The authors note that subjectively, it was quite comfortable to have their fundus photos taken with the prototype camera compared to indirect ophthalmoscopy, which suggests an additional advantage of nonmydriatic imaging.

Our prototype, or a camera based on our prototype, may have myriad uses for health professionals. For example, it may be useful for ophthalmologists seeing inpatient consults, as moving inpatients to a stationary fundus camera can be impractical, and many neurosurgery inpatients in the intensive care unit are not allowed to be pharmacologically dilated. The comfort of nonmydriatic imaging may make the camera useful for pediatric ophthalmologists, although alignment might be difficult. Finally, the low cost and small size of our camera may make the camera a valuable tool for ophthalmologists practicing global medicine. With added features such as a large memory card and a strong wireless card or cell phone antenna, the device could help providers practice telemedicine.

We are making the instructions for this device open source (see Supplementary Instructions), with the hope that others will build it and make further modifications in an innovative way.

## 5. Conclusion

Our prototype camera takes advantage of recent advances in camera and LED technology and is a proof of concept that nonmydriatic fundus cameras do not need to be bulky and expensive. Using almost entirely off-the-shelf electronic parts, we were able to construct a handheld nonmydriatic camera weighing 386 grams and costing $185.20, which is much lighter and less expensive than currently available cameras. With additional refinements and additional safety testing, our prototype camera, or a camera based on our prototype, may offer the advantages of nonmydriatic fundus imaging to a wider range of medical professionals.

## Supplementary Material

The Supplementary Documents includes both instructions on building the camera, as well as a parts list.
