# Supplementary material for: A Portable, Inexpensive, Nonmydriatic Fundus Camera Based on the Raspberry Pi® Computer
Source: J Ophthalmol. 2017 Mar 15;2017:4526243. doi: 10.1155/2017/4526243 (PMC5370517; doi:10.1155/2017/4526243)
Supplement: Supplementary file 1 — The Supplementary Documents includes both instructions on building the camera, as well as a parts list. [file 4526243.f1.docx]

**Step-by-step instructions for programming and assembling the camera:**

***All websites accessed on 2/24/2016***

1. Set up the Raspberry Pi 2 and install the Raspbian operating system onto the Raspberry Pi 2 using the NOOBS microSD card, an external monitor, an HDMI cable, an external USB keyboard and mouse, and a power adapter. Detailed instructions can be found here: <https://www.raspberrypi.org/help/videos/>.
2. Change the settings on the Raspberry Pi 2 so that the Raspberry Pi 2 boots directly to the desktop per the instructions here: <https://www.raspberrypi.org/documentation/configuration/raspi-config.md>.
3. Set up the Adafruit 5’’ HDMI Backpack on the Raspberry Pi 2 using the instructions here: <https://learn.adafruit.com/adafruit-5-800x480-tft-hdmi-monitor-touchscreen-backpack>.
4. Charge the Mogix external battery with a power adapter, and connect the battery to the HDMI Backpack and the Raspberry Pi 2 using micro-USB cables.
5. Connect the HDMI Backpack to the Raspberry Pi 2 using an HDMI cable. Now when the Raspberry Pi 2 is powered on, the HDMI Backpack will be the Raspberry Pi 2’s display.
6. Set up the NoIR camera board per the instructions found here: <https://www.raspberrypi.org/learning/getting-started-with-picamera/>.
7. Turn off the camera board LED using the instructions found here: <http://www.raspberrypi-spy.co.uk/2013/05/how-to-disable-the-red-led-on-the-pi-camera-module/>.
8. Using two pairs of jewelry pliers, adjust the focus of the NoIR camera board to about 8 cm in front of the camera as per <https://www.raspberrypi.org/learning/infrared-bird-box/>.
9. Write the following script in Python 2, and save it on the Raspberry Pi 2:

import time

import picamera

import RPi.GPIO as GPIO

GPIO.setmode(GPIO.BCM)

GPIO.setup(17, GPIO.IN, GPIO.PUD_UP)

*# GPIO 17 is the shutter button*

GPIO.setup(22, GPIO.OUT)

*# GPIO 22 is the infrared cathode*

GPIO.setup(23, GPIO.OUT)

*# GPIO 23 is the white light cathode*

GPIO.output(22, False)

*# Sets the infrared cathode to ground. The common anode of the*

*# LED is set to 3.3 V, so setting GPIO 22 to ground causes the*

*# infrared light to be on.*

GPIO.output(23, True)

*# Sets the white light cathode to 3.3 V*

with picamera.PiCamera() as camera:

camera.start_preview()

GPIO.wait_for_edge(17, GPIO.FALLING)

GPIO.output(22, True)

GPIO.output(23, False)

camera.capture('/home/pi/Desktop/image1.jpg', use_video_port=True)

camera.stop_preview

GPIO.cleanup()

This is the non-mydriatic fundus camera program. It will save a picture called “image1” on the desktop when the shutter button is pressed.

1. Solder each of the three terminals on the SMT47W/850D LED chip to individual 6’’ female/female jumper cables. The top right terminal is the common anode, and can be connected to a 3.3 V GPIO pin on the Raspberry Pi 2 (e.g., physical pin #1). The bottom left terminal controls the white light, and can be connected to GPIO pin #23 (physical pin #16). The bottom right terminal controls the infrared light; connect this terminal to a 100-ohm resistor, and connect the resistor to GPIO pin #22 (physical pin #15). For more details on GPIO pins, please see <https://www.raspberrypi.org/documentation/usage/gpio-plus-and-raspi2/>.
2. Connect one side of a tactile switch button to one of the ground GPIO pins (e.g., physical pin #6), and the other side to GPIO pin #17. This switch button will act as the shutter button.
3. Use rubber bands and electrical tape to assemble the camera as per Figures 1 and 2. I taped the shutter button on the top of the battery. I used rubber bands to keep the camera together, with the Mogix battery sandwiched in between the Raspberry Pi 2 and HDMI Backpack.
4. Install the Florence virtual keyboard by opening a command window and entering:

sudo apt-get update
sudo apt-get install Florence

You can launch the Florence virtual keyboard from “Menu”->”Universal Access”.

1. To operate the camera, plug the micro-USB cable into the Raspberry Pi 2 power port, which will turn on the Raspberry Pi 2. The Raspberry Pi 2 should boot up directly into the desktop. Use the HDMI Backpack’s touchscreen to open a command window. Open the Florence virtual keyboard from the desktop menu, and type “sudo idle &” into the command window, which will open the Python 2 program. Open the non-mydriatic fundus camera program, and run it.
